# Crystal Structure of the FERM-SH2 Module of Human Jak2

**DOI:** 10.1371/journal.pone.0156218

**Published:** 2016-05-26

**Authors:** Randall McNally, Angela V. Toms, Michael J. Eck

**Affiliations:** 1 Department of Cancer Biology, Dana-Farber Cancer Institute, Boston, Massachusetts, United States of America; 2 Department of Biological Chemistry and Molecular Pharmacology, Harvard Medical School, Boston, Massachusetts, United States of America; Yale University School of Medicine, UNITED STATES

## Abstract

Jak-family tyrosine kinases mediate signaling from diverse cytokine receptors. Binding of Jaks to their cognate receptors is mediated by their N-terminal region, which contains FERM and SH2 domains. Here we describe the crystal structure of the FERM-SH2 region of Jak2 at 3.0Å resolution. The structure reveals that these domains and their flanking linker segments interact intimately to form an integrated structural module. The Jak2 FERM-SH2 structure closely resembles that recently described for Tyk2, another member of the Jak family. While the overall architecture and interdomain orientations are preserved between Jak2 and Tyk2, we identify residues in the putative receptor-binding groove that differ between the two and may contribute to the specificity of receptor recognition. Analysis of Jak mutations that are reported to disrupt receptor binding reveals that they lie in the hydrophobic core of the FERM domain, and are thus expected to compromise the structural integrity of the FERM-SH2 unit. Similarly, analysis of mutations in Jak3 that are associated with severe combined immunodeficiency suggests that they compromise Jak3 function by destabilizing the FERM-SH2 structure.

## Introduction

Jak-family tyrosine kinases transmit extracellular signals from diverse cytokine receptors and regulate a variety of cellular processes including immune responses, differentiation, hematopoiesis, and growth. The four family members (Jak1, Jak2, Jak3, and Tyk2) associate with the cytoplasmic domain of specific cytokine receptors, and receptor engagement results in activation of associated Jak kinases, which phosphorylate and activate STAT transcription factors [1,2]. Jak2, for example, binds and signals from a number of Type I and Type II cytokine receptors, including those for erythropoietin (EPO), thrombopoietin (TPO), growth hormone, and prolactin. These receptors are all homodimeric, and thus engage two copies of Jak2. Jak2 can also heterodimerize with Jak1 or Tyk2 when associated with heterodimeric cytokine receptors, including those for interleukin (IL)-3, IL-5, IL-12 and interferon-γ. Suppression of Jak function or its pathway can result in compromised immune function, while constitutive activation of Jak kinases by point mutations or chromosomal translocations can result in myeloproliferative disease or cancer [3].

**Fig 1:**
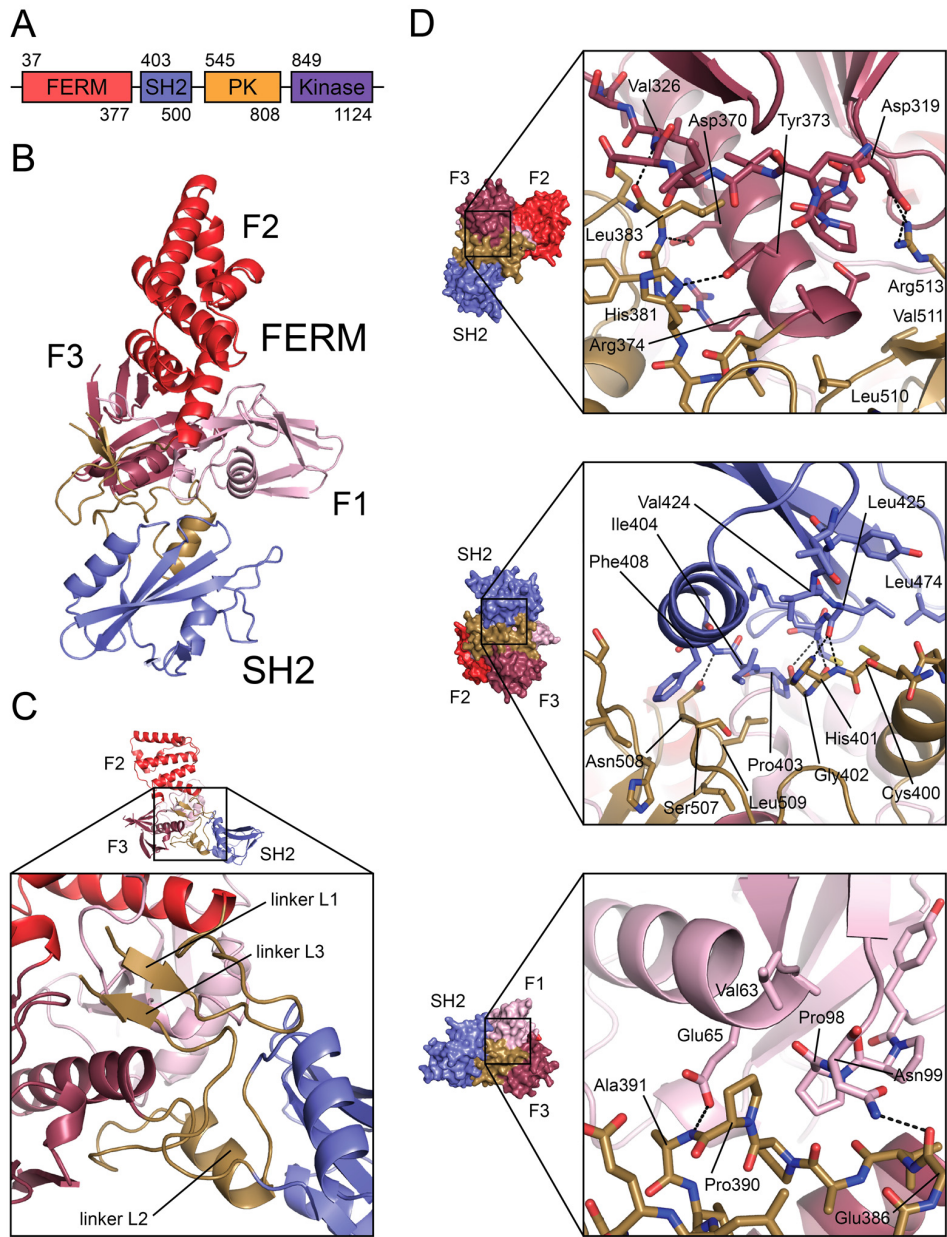
Crystal structure of the FERM-SH2 domains of Jak2. (A) The domain structure of Jak-family kinases; residue numbers correspond to human Jak2. ‘SH2’, SH2-like. (B) Structure of Jak2 FERM-SH2 domains, with domains and sub-domains indicated. (C) Detail of Jak-specific insertions, linkers L1, L2, and L3. L1 connects FERM F1 and F2 lobes; linker L2 connects FERM F3 lobe and SH2 domain; linker L3 connects SH2 and pseudokinase domains. (D) Interfaces between Jak-specific insertions and FERM, SH2 domains. Top panel: FERM F3 lobe (dark red) and linkers L2 and L3 (gold). Center panel: SH2 domain (blue) and linkers L2 and L3. Bottom panel: FERM F1 lobe (pink) and linker L2. Dashed lines indicate hydrogen bonds and salt bridges across interfaces. Crystal structure figures were generated using PyMOL.

Jak proteins contain four structural domains: an N-terminal FERM (Band 4.1, Ezrin, Radixin, Moesin-homology) domain, an SH2 domain, a pseudokinase domain, and a C-terminal catalytic (kinase) domain (Fig 1A). The FERM and SH2 domains are necessary for the interaction with the cytoplasmic tails of the receptors, which contain “box1” and “box2” motifs required for Jak engagement. Box1 is proline-rich while box2 usually consists of a negatively-charged residue followed by several hydrophobic residues. The two motifs are poorly conserved between receptors, but are necessary for proper Jak activity across Jak-receptor pairs [4,5]. The FERM domain of Jak2 is also important for its proper catalytic regulation; deletion or mutation of this region results in increased basal kinase activity [6]. The Jak SH2 domain has been shown to be necessary for receptor interaction and Jak activation, but it is not believed to maintain the phosphotyrosine-binding function of classical SH2 domains [7,8]. The pseudokinase domain is thought to serve primarily a regulatory role via interactions with the adjacent tyrosine kinase domain [9–12]. In Jak2, a V617F mutation within the pseudokinase domain leads to constitutive activation of the kinase and gives rise to myeloproliferative disorders including polycythemia vera [13].

A recent structure of the FERM-SH2 region of Tyk2 revealed that the two domains form a tightly integrated structural module [14]. The structure was determined in complex with a fragment of interferon-α receptor 1 (IFNAR1), the α-chain of the receptor for the interferons –α and –β. The structure showed that the box2 region of IFNAR1 binds to the SH2 domain in a manner strikingly reminiscent of the phosphopeptide interaction with SH2 domains [15–17]. In this structure, a glutamic acid residue in the IFNAR1 box2 motif inserts into the canonical phosphotyrosine pocket, and a hydrophobic segment C-terminal to the glutamic acid lies in a specificity-conferring groove in the SH2 domain. Tyk2 is the only Jak family member for which structural information for the FERM-SH2 region is available, thus it remains unclear how the structure of this region varies among family members and how specificity for specific subsets of cytokine receptors is achieved.

To better understand the role of the FERM-SH2 region in Jak2 function, as well as to identify potential determinants of specificity of Jak family members for their cognate receptors, we determined the X-ray crystal structure of the Jak2 FERM-SH2. Analysis of the structure reveals an overall similarity to the corresponding region of Tyk2 as well as some conformational differences, and we identify differences in the receptor binding site between Jak2 and Tyk2 that may contribute to specificity of receptor binding. We also use the Jak2 structure to demonstrate how Jak3 mutations identified in patients result in severe combined immunodeficiency (SCID).

## Materials and Methods

### Expression and purification

Jak2 FERM-SH2 (residues 32–516) was cloned into a modified pTriEx-1.1 vector (Novagen) with a C-terminal 8-His tag appended. Recombinant baculovirus was generated using the BacVector3000 system (Invitrogen). *Trichoplusia ni* (Hi-5) cells at a density of 1.4x10^6^ cells/mL were infected using the Titerless and Infected-Cells Preservation and Scale-Up (TIPS) method [18] and induced at 27°C for 55–60 hours. Cells were harvested by centrifugation and resuspended in 20 mM Tris pH 7.5, 500 mM NaCl, 20 mM imidazole, 10% glycerol, and 5 mM β-mercaptoethanol.

Resuspended cells were lysed by the addition of 1% Nonidet-P40 followed by incubation on ice for 40 minutes. Following centrifugation at 17,000 RPM for 40 minutes to remove cellular debris, lysate was applied to Ni-NTA agarose resin (Qiagen), washed with resuspension buffer, and eluted with resuspension buffer that contained 250 mM imidazole. The eluate was diluted in SP buffer (containing 10 mM Na_2_HPO_4_ pH 7.0, 2 mM KH_2_PO_4_, 10% glycerol, 4 mM) to a final NaCl concentration of 250 mM and applied to a SP Sepharose HP 5 mL column (GE Healthcare). FERM-SH2 was eluted in SP buffer with a NaCl gradient ranging from 250 mM to 1M NaCl over 20 column volumes. The eluted product was concentrated and purified over a Superdex 200 10/300 GL column (GE Healthcare) in 20 mM Tris pH 7.5, 250 mM NaCl, and 4 mM DTT. Selected fractions were concentrated to 5–10 mg/mL, frozen in liquid nitrogen, and stored at -80°C.

### Crystallization and structure determination

Crystals of Jak2 FERM-SH2 were prepared using the hanging drop vapor diffusion method; an equal volume of well solution (0.2 M Na Citrate, 11–13% PEG 3350, 1.05% 1-butanol, 5 mM TCEP) was added to Jak2 FERM-SH2 and equilibrated over well solution at 20°C. Crystals were looped into cryoprotectant solution (0.2 M Li Citrate, 20 mM Tris pH 7.5, 125 mM NaCl, 1.05% 1-butanol, 20% PEG 3350, 5% glycerol, 5 mM TCEP) for 30–60 seconds, then flash-frozen in liquid nitrogen.

X-ray diffraction data were collected at APS beamlines 19-ID (Structural Biology Center) and 24-ID-C (NE-CAT), processed with XDS [19], and scaled with Scala [20,21]. The structure was solved by molecular replacement with Phaser [22] using the Tyk2 FERM-SH2 domains as a search model (PDB ID 4PO6) [14]. The crystallographic model was manually built using Coot [23] into an 8-fold NCS-averaged map calculated by Phenix Autobuild [24], and refined with Phenix [24] and BUSTER [25].

Coordinates and structure factors have been deposited in the Protein Data Bank under accession code 4Z32.

### Size-exclusion chromatography/multi-angle light scattering analysis

Jak2 FERM-SH2 (750 μg) was applied to a Superdex 200 10/300 GL column (GE Healthcare) in 20 mM Tris pH 7.5, 250 mM NaCl, 0.02% CHAPS, and 4 mM DTT. In-line multi-angle light scattering analysis was performed with an OptiLab rEX refractive index detector followed by a miniDAWN TREOS light scattering detector, and data were analyzed with ASTRA (Wyatt Technology).

### Surface area analysis

Surface area buried within interfaces between Jak2 FERM-SH2 monomers in the crystallographic asymmetric unit was calculated using the PDBePISA server [26].

### Structural alignments

Structurally equivalent residues between Jak2 and Tyk2 FERM-SH2 were defined using the Dali Server pairwise comparison [27]. The matching residues were input into Superpose [28] to align Jak2 and Tyk2 and to calculate the rmsd and displacements between equivalent Cα atoms. Superpose was used to calculate the rmsd for Cα atoms between Jak2 FERM-SH2 monomers within the crystallographic asymmetric unit.

### Sequence conservation analysis

Sequence alignments and calculation of conservation scores were performed using The ConSurf Server [29]. The UNIREF-90 database was searched for homolog sequences of the input Tyk2 and Jak2 FERM-SH2 structures using the CSI-BLAST algorithm, with three iterations, an E-value cutoff of 0.0001, and 35% minimal ID for homolog sequences. The sequence alignment was built using MAFFT.

## Results and Discussion

### Structure determination

We reproducibly obtained crystals of the FERM-SH2 module that diffracted to approximately 3.0 Å resolution, but experimental phasing of the structure proved problematic. Extensive efforts with heavy atom soaking were unsuccessful, and we were unable to obtain sufficient phasing power using MAD or SAD approaches with selenomethionine-incorporated protein produced in insect cells, perhaps due to a combination of modest selenium incorporation (~70%) and the high non-crystallographic symmetry present in the crystals. Likewise, molecular replacement with diverse FERM and SH2 domains of known structure was unsuccessful. However, the recent elucidation of the structure of the corresponding region of Jak-family member Tyk2 allowed straightforward structure determination by molecular replacement, which revealed eight copies of the FERM-SH2 module in the asymmetric unit. Within the asymmetric unit, the FERM-SH2 molecules are arrayed as a compact module with approximate 422 point symmetry (S1A Fig). Despite the considerable surface area buried in this oligomer, with each monomer burying approximately 1900 Å^2^ with its neighbors within the asymmetric unit, it is unlikely to be biologically relevant. The purified protein is monomeric as assessed by size-exclusion chromatography combined with multi-angle light scattering (SEC/MALS, S1B Fig).

The eight-fold non-crystallographic symmetry allowed for phase improvement by iterative NCS averaging in Phenix [24]. Maps calculated with the resulting phases were free of any apparent model bias, and allowed straightforward rebuilding of the model in well-ordered regions, even in areas of dramatic deviation from the starting Tyk2 model (S2 Fig). The eight molecules in the asymmetric unit are closely similar, though some of the molecules are better ordered than others. Molecule B of our FERM-SH2 structure had the lowest average temperature factor and most complete electron density of the eight molecules in the asymmetric unit, and thus further discussion of the Jak2 FERM-SH2 structure will refer to this molecule. The rmsd for Cα atoms between the “B” molecule and the seven other molecules ranges from 0.31–0.52 Å. The final model included residues 37–514, and was refined to a crystallographic R value of 25.7% (R_free_ = 27.6%) at 3.0Å resolution (Table 1).

**Table 1:** Data collection and refinement statistics.

**Data Collection**	
Space group	P 21
Cell dimensions	
*a*, *b*, *c* (Å)	118.19, 188.74, 118.59
*α*, *β*, *γ* (°)	90, 113.87, 90
Resolution (Å)	3.0 (3.19–3.04)^a^
R_sym_	0.075 (0.534)
*I/σI*	9.5 (2.0)
Completeness (%)	96.4 (98.9)
Redundancy	2.1 (2.1)
**Refinement**	
Resolution (Å)	43.0–3.0
No. reflections	87695
R_work_/R_free_ (%)	25.7/27.6
No. atoms	
Protein	27917
Ligand/ion	0
Water	12
*B* factors	
Protein	88.2
Water	35.4
r.m.s. deviations	
Bond lengths (Å)	0.011
Bond angles (°)	1.603

^a^Values in parentheses are for highest-resolution shell

### Overall structure of Jak2 FERM-SH2

The Jak2 FERM-SH2 crystal structure reveals a compact module in which the SH2 domain is intimately associated with the FERM domain (Fig 1B). The FERM region maintains the typical three-lobed architecture, with an F1 lobe consisting of a ubiquitin-like fold, an F2 lobe consisting of an acyl-CoA binding protein fold, and an F3 lobe consisting of a pleckstrin-homology (PH) fold [30,31]. The SH2 region interacts with both the F1 and F3 lobes, and in addition, the interdomain linker segments interact with each other and with the FERM and SH2 elements to lace the domains into an integrated structural unit (Fig 1C). As described below, the overall architecture is the same as that observed in the Tyk2 structure, and we adopt the same naming convention for the intervening linker segments (linker L1 connects the F1 and F2 lobes, linker L2 connects the F3 lobe and SH2 domain, and linker L3 follows the SH2 domain).

The linker segments contribute extensive polar and hydrophobic contacts in joining domains and sub-domains in the structure. The FERM F3 lobe and SH2 domain sandwich linkers L2 and L3. A troika of hydrogen bonds contributed by backbone atoms of linker L2 residues His381 and Leu383 span the interface with FERM F3, while Arg513 of linker L3 forms a salt bridge with FERM F3 residue Asp319 (Fig 1D, top panel). In addition, Leu383 and linker L3 residues Leu510 and Val511 are buried in the interface with FERM F3 (Fig 1D, top panel). The interface of linkers L2 and L3 with the SH2 domain is also stabilized by a number of hydrogen bonds with backbone atoms, contributed by Ser405, Leu425, and Cys427 of the SH2 domain and His401 and Gly402 of linker L2 (Fig 1D, center panel). Buried hydrophobic residues in this interface include Pro403, Ile404, and Phe408 of the SH2 domain, Cys400 of linker L2, and Leu509 of linker L3 (Fig 1D, center panel).

Further, linker L2 forms contacts with the FERM F1 lobe. Backbone atoms belonging to L2 residues Glu386 and Ala391 hydrogen bond with Asn99 and Glu65, respectively, of the FERM F1 lobe (Fig 1D, bottom panel). Also, Pro390 of linker L2 and Val63 and Pro98 of the F1 lobe are buried in the interface (Fig 1D, bottom panel).

### Comparison with Tyk2 FERM-SH2

**Fig 2:**
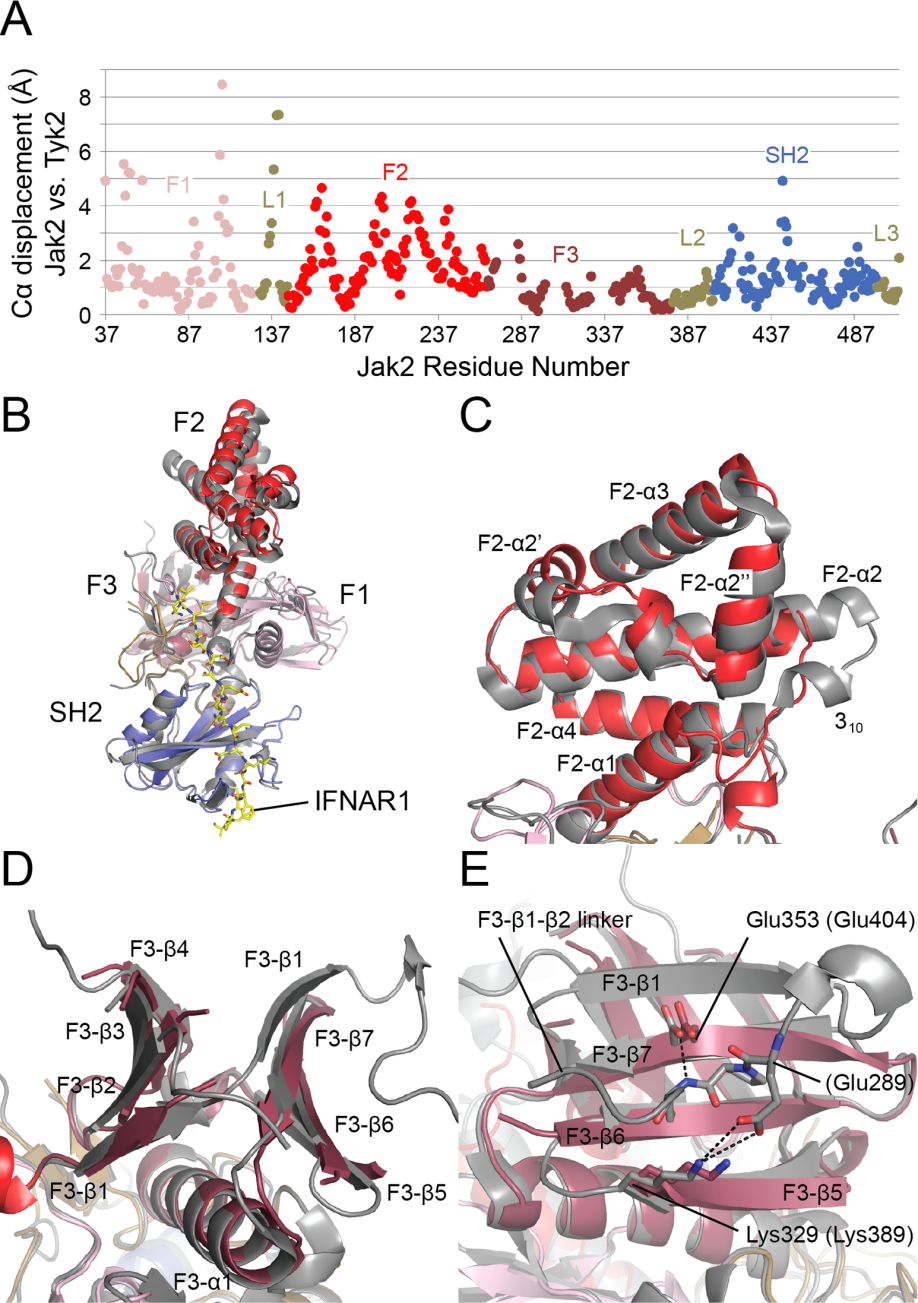
Comparison of the structures of Jak2 and Tyk2-IFNAR1. (A) Plot of the displacement of structurally equivalent Cα atoms between superposed structures of Jak2 and Tyk2. (B) Superposition of Jak2 (colored as in Fig 1) and Tyk2-IFNAR1 (Tyk2 is colored gray, IFNAR1 is yellow). (C-E) Detailed views of superposition in (B). (C) The FERM F2 lobe of Jak2 differs from that of Tyk2. The linker between helices F2-α1 and F2-α2 is unstructured in Jak2, while in Tyk2 it forms a 3_10_ helix and N-terminal extension of helix F2-α2. In addition, helices F2-α2’, F2-α2”, and F2-α3 of the Jak2 F2 lobe are displaced relative to Tyk2. (D-E) The FERM F3 lobe of Jak2 differs from that of Tyk2. A C-terminal extension of strand F3-β1 and subsequent linker that forms further interactions with the F3 lobe in Tyk2 are absent in the Jak2 structure. Dashed lines indicate hydrogen bonds and salt bridges. Jak2 residues are labeled with corresponding Tyk2 residues in parentheses.

The global architecture of the Jak2 and Tyk2 FERM-SH2 structures are closely similar (Fig 2B); they exhibit the same relative orientation of the FERM and SH2 regions, and the L1, L2 and L3 linkers adopt the same conformation and role in both structures. The Tyk2 structure included an IFNAR1 receptor fragment, but the presence of the bound receptor does not appear to have altered domain orientations, despite the fact that it bridges between the SH2 and FERM regions. Despite the similar domain orientations, the two structures exhibit marked deviations in several loop regions as well as within the F2 lobe of the FERM domain (Fig 2A and S3 Fig). Overall, the structures superimpose with an rmsd of 1.87 Å for 427 structurally equivalent Cα atoms.

The structures differ most significantly in the F2 lobe (Fig 2C). A short linker between α-helices F2-α1 and F2-α2 (using secondary structure labeling convention established for the FAK FERM domain [30]) in Jak2 becomes a 3_10_ helix and one-turn N-terminal extension of helix F2-α2 in Tyk2; this difference displaces equivalent Cα atoms within α-helices F2-α2’, F2-α2”, and F2-α3 up to 4.3 Å relative to Tyk2 (Fig 2C). Another structural difference between Jak2 and Tyk2 occurs in the F3 lobe at strand F3-β1 and subsequent linker, where Jak2 has a 23-residue deletion relative to Tyk2. In Tyk2, strand F3-β1 is 12 residues long and it hydrogen bonds with both strand F3-β2 and strand F3-β7 (Fig 2D). Furthermore, the connecting loop between strands F3-β1 and F3-β2 makes additional interactions with the F3 lobe (Fig 2E). In the Jak2 structure, however, these features are largely absent due to the 23-residue deletion; only the five N-terminal residues of strand F3-β1 are preserved (Fig 2D and 2E).

**Fig 3:**
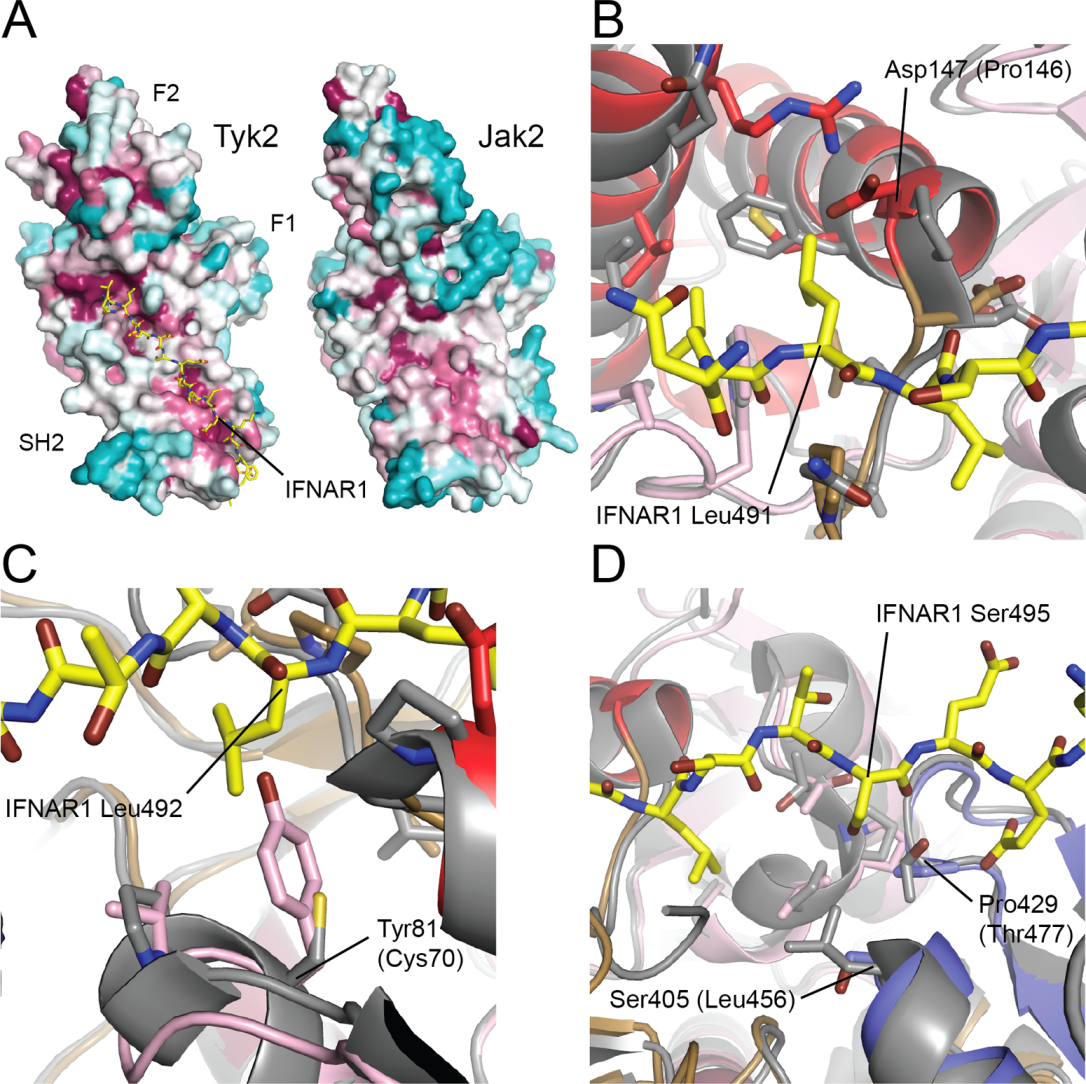
Insights into the specificity of receptor binding to Jak2 vs. Tyk2. (A) Sequence conservation among Jak family members across phylogeny, mapped onto the surfaces of Tyk2 and Jak2 FERM-SH2. The surface is shaded from magenta (most conserved) to teal (most variable) on the basis of ConSurf analysis. The surface of Jak2 that corresponds to the highly conserved IFNAR1 binding pocket on Tyk2 is also highly conserved, suggesting that the receptor-binding function of this surface is retained for Jak2. (B-D) Superposition of the structures of Jak2 (colored as in Fig 1) and Tyk2-IFNAR1 (Tyk2 is colored gray, IFNAR1 is yellow) reveal differences that may determine the specificity of receptors that Jak2 and Tyk2 are capable of binding. (B) In the IFNAR1 Leu491 binding pocket of Tyk2, Pro146 is replaced by Asp in Jak2, a charged residue that is incompatible with the hydrophobic IFNAR1 Leu491. (C) In the IFNAR1 Leu492 binding pocket of Tyk2, replacement of Tyk2 Cys70 with Tyr (as in Jak2) results in steric occlusion of IFNAR1 Leu492. (D) In the IFNAR1 Ser495 binding pocket of Tyk2, the polar Tyk2 Thr477 is replaced in Jak2 by the hydrophobic Pro429, while in the base of the pocket the bulky, hydrophobic Tyk2 Leu456 is replaced by the relatively small, polar Ser405 in Jak2.

An obvious question of interest is how particular Jak family members engage specific cytokine receptors. We have not been able to obtain co-crystals with receptor fragments, and even binding studies with fragments of both the EPO and TPO receptor tails and the Jak2 FERM-SH2 fragment have proven frustratingly inconclusive, perhaps due to a requirement for the lipid bilayer in the interaction [32]. However, analysis of conservation of Jak2 residues using ConSurf reveals that the surface of Jak2 that is structurally equivalent to the IFNAR1 fragment binding site in Tyk2 is similarly highly conserved (Fig 3A), indicating that the receptor-binding function of this surface is also likely conserved in Jak2. Thus, comparison with the Tyk2/IFNAR1 structure provides some insight into likely determinants of receptor specificity. For example, Tyk2 Pro146 in the IFNAR1 Leu491 binding pocket is replaced with an aspartic acid in Jak2 (Fig 3B), which is likely incompatible with a receptor with a bulky hydrophobic residue in that position. Similarly, Jak2 Tyr81 (equivalent to Cys70 in Tyk2) is modeled to sterically interfere with Leu492 of IFNAR1 (Fig 3C). Finally, Tyk2 Leu456 and Thr477 define the IFNAR1 Ser495 binding pocket, while these residues are replaced, respectively, with Ser405 and Pro429 in Jak2 (Fig 3D); these changes may allow Jak2 to accommodate a larger residue in this position from a bound receptor.

### The canonical phosphotyrosine pocket is blocked in the Jak2 SH2 domain

**Fig 4:**
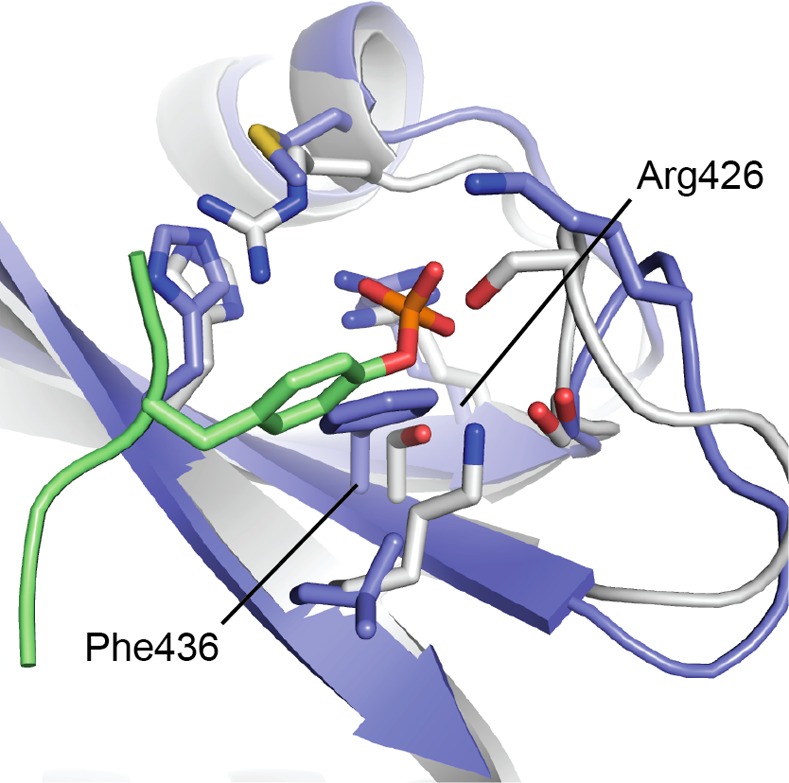
The Jak2 SH2 domain lacks a typical phosphotyrosine-binding pocket. The crystal structure of the SH2 domain of the Src-family kinase Lck bound to a high-affinity phosphotyrosyl peptide (PDB ID 1LCJ) [33] is superimposed on the Jak2 SH2 domain. The Lck structure is shown as a white ribbon, with the bound phosphopeptide in green. The Jak2 SH2 domain is colored blue. The phosphotyrosine sidechain and selected residues in the binding pocket are shown in stick form. Note that Phe436 in the Jak2 SH2 domain blocks the position that would be occupied by the phenyl group of a bound phosphotyrosine. Labels refer to Jak2 residues.

The SH2 domain in Jak2 and other family members is widely thought to lack phosphotyrosine-binding function, in part because early studies found no phenotype with mutation of a key conserved arginine in this domain [4,8]. Consistent with these studies, the present structure suggests that the Jak2 SH2 domain is incapable of phosphotyrosine recognition, at least in the general manner observed for other SH2 domains. Superposition of this region of the Jak2 SH2 domain on that of Lck, a Src-family member that exhibits the prototypical mode of phosphotyrosine binding, reveals that the phosphotyrosine binding site is blocked in the Jak2 SH2 domain (Fig 4). Although Jak2 retains the key arginine residue required for phosphotyrosine coordination (Arg426), as well as additional basic residues in this region, a phenylalanine residue (Phe436) blocks the portion of the pocket that would normally accommodate the phenyl ring of a phosphotyrosine residue. In SH2 domains that retain the ability to recognize phosphotyrosine, a small polar or hydrophobic residue occupies this position (serine, threonine, alanine, or valine). By contrast, Jak-family SH2 domains have a large hydrophobic residue in this position (phenylalanine in Jak2, leucine in Jak1 and Jak3, and isoleucine in Tyk2).

### FERM mutations that block receptor association cluster in the hydrophobic core

**Fig 5:**
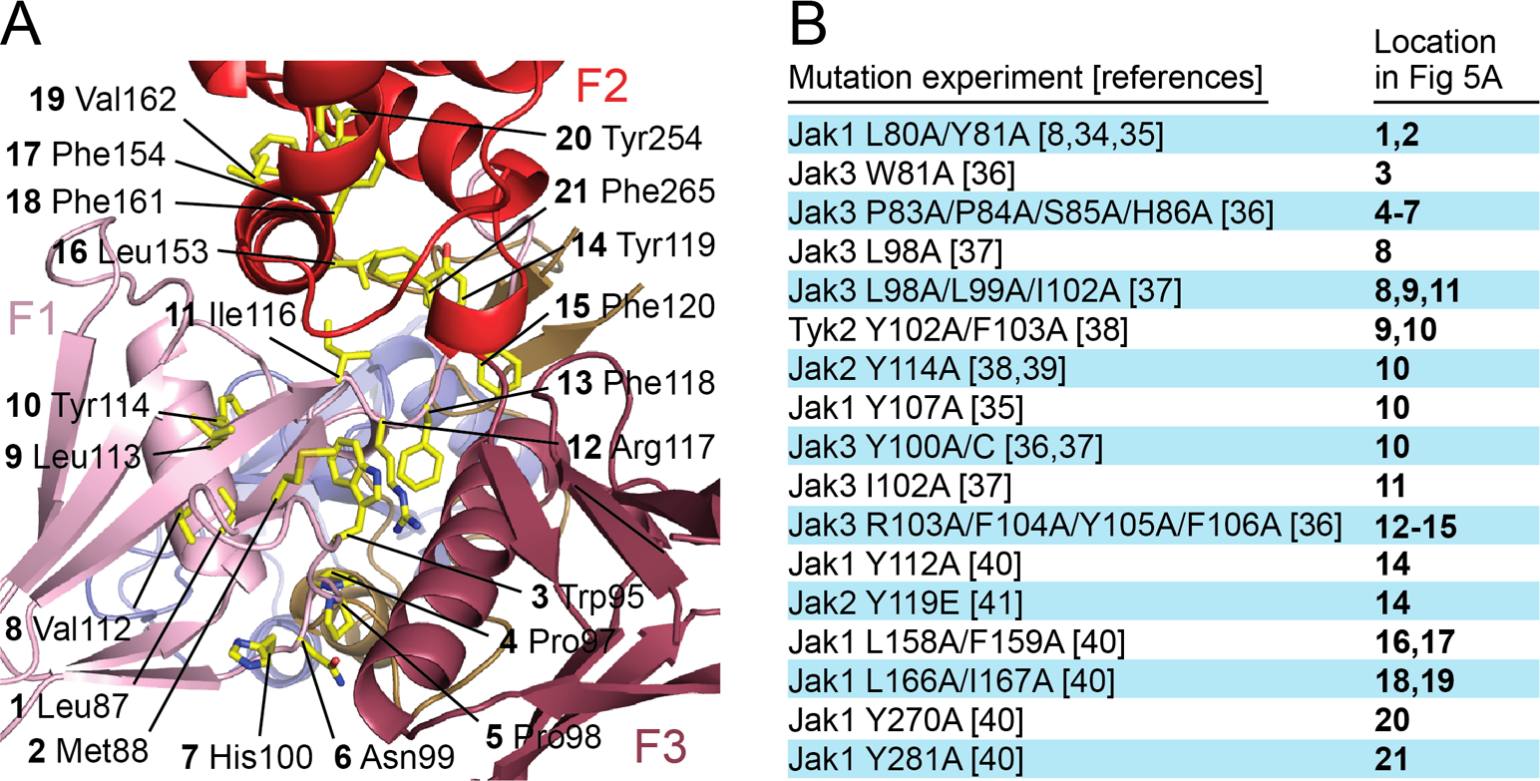
Functional mutations in Jak mapped onto the structure of Jak2 FERM-SH2. (A) Residues in the Jak FERM domain that, when mutated, have been found experimentally to result in deficient binding to cytokine receptor are mapped to their corresponding residues on Jak2 and colored yellow. These residues are largely buried, indicating that the effect on receptor binding from mutating them is likely a result of destabilization of the FERM domain. Labels indicate Jak2 equivalents of mutated residues. (B) List of mutation experiments referred to in (A). Not pictured: SCID mutants Jak3 delA58 (equivalent to Jak2 Ser72), Jak3 D169E (equivalent to Jak2 Asp185), and Jak3 R402H (equivalent to Jak2 Arg426) [36,42].

The four Jak family members have been the subject of numerous structure/function studies over the past two decades, including many studies that report mutations in the FERM domain that interfere with receptor association. In addition, a number of disease-associated mutations have been found in the FERM region. We tabulated mutations reported to disrupt receptor binding in one or more Jak family members [8,34–42] and mapped them on the Jak2 FERM-SH2 structure, with the hope of identifying potential sites of receptor interaction (Fig 5). Strikingly, these mutations lie almost exclusively in the hydrophobic core of the FERM domain. Almost all are buried hydrophobic residues or charged residues that form a buried salt bridge interaction (for example Arg117, which forms a buried salt bridge with Glu268), or are exposed but mutated in conjunction with buried hydrophobic residues (as is the case for Asn99/His100, mutated along with buried residues Pro97/Pro98). Thus, we expect that mutation of these residues results in loss of function and receptor binding because they compromise the structural integrity of the FERM domain, and not due to a specific role in receptor binding or Jak autoregulation.

### Structural effects of disease-associated mutations

**Fig 6:**
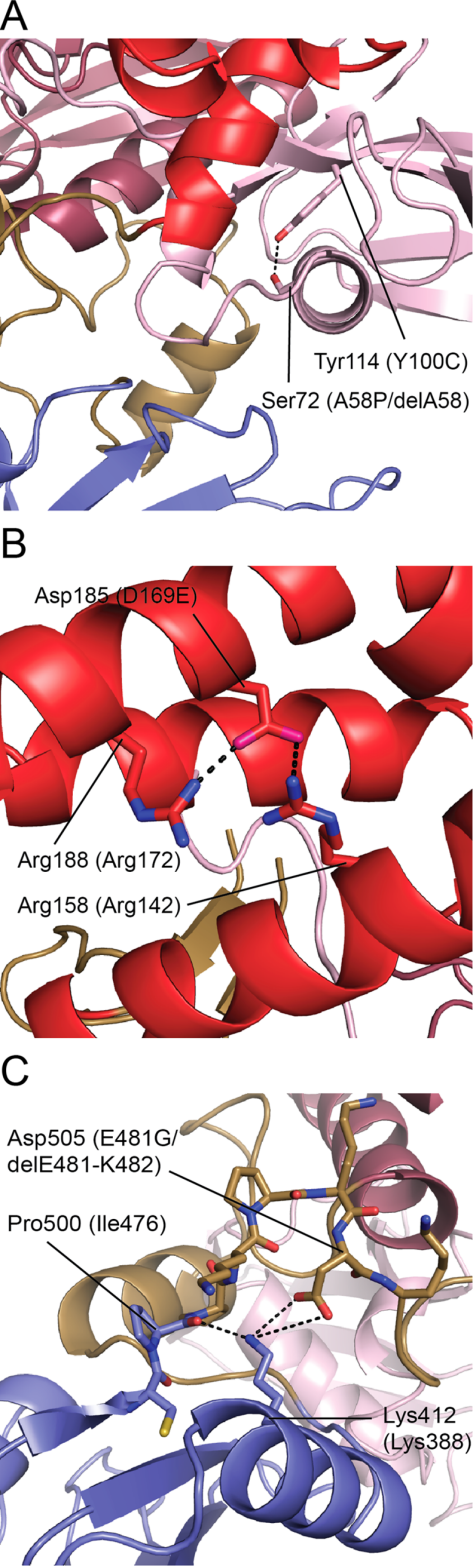
Jak3 SCID mutations mapped onto structure of Jak2. (A) Jak2 residues Tyr114 and Ser72, equivalent in position to SCID mutations Jak3 Y100C and A58P, respectively, are buried in the FERM F1 lobe. These mutations, along with SCID mutation Jak3 delA58, likely destabilize the F1 lobe. (B) Jak2 Asp185 makes salt bridge interactions with Arg158 and Arg188. The SCID mutation Jak3 D169E (equivalent in position to Jak2 Asp185) potentially disrupts this structural network. (C) The Jak2 residue Asp505, equivalent in position to SCID mutation Jak3 E481G, participates in a structural network across the L3-SH2 interface with Lys412 and the backbone carbonyl of Pro500. This mutation, as well as SCID mutation delE481-K482, disturbs this network. Dashed lines indicate hydrogen bonds and salt bridges. Jak2 residues are labeled with corresponding Jak3 residues in parentheses.

The Jak2 structure also allows us to rationalize the structural effects of several Jak3 mutations in patients with severe combined immunodeficiency (SCID). We mapped SCID-associated mutations within the FERM-SH2 region of Jak3 to their analogous locations on the Jak2 FERM-SH2; analysis of these mutations indicates that they likely prevent proper Jak3 function by disrupting the stability of Jak3 FERM-SH2. Three of these mutations, Y100C [43,44], A58P [45], and delA58 [36] occur in the F1 lobe (Fig 6A). Y100C (equivalent to Jak2 Tyr114), replaces a buried tyrosine. A58P (Jak2 Ser72) is near Tyr100, and is expected to lie within an alpha helix. This mutation, by placing a proline within a helix, would likely destabilize the helix and thus the F1 lobe. Similarly, the delA58 mutation, which deletes one residue and therefore shifts the register of this helix, would also be expected to destabilize the fold of the domain.

A SCID mutation in the F2 lobe, D169E, potentially disturbs a structural network; its equivalent residue in Jak2 (Asp185) is buried and makes polar contacts with Arg158 and Arg188 (Jak3 equivalents Arg142 and Arg172, respectively) (Fig 6B). When transfected into COS-7 cells, Jak3 D169E, Y100C, and delA58 were diminished in both in vitro kinase activity and ability to immunoprecipitate with a chimeric receptor containing the cytoplasmic segment of the common γ chain (γ_c_) [36].

The Jak2 structure predicts that Jak3 SCID mutations E481G [43,45,46] and delE481-K482 [46,47] in linker L3 would destabilize the L3-SH2 interface. Jak2 Asp505, equivalent in position to Jak3 E481G, forms a salt bridge with Lys412 (Lys388 in Jak3) across the L3-SH2 interface (Fig 6C).

Finally, an immunodeficiency patient-derived mutation in the Jak3 SH2 domain, R402H, fails to promote membrane expression of γ_c_ [42]. This residue is analogous to Tyk2 His474, a critical element of the IFNAR1 box2 binding site [14]. The functional deficiency resulting from this mutation may thus be a consequence of defective receptor binding; though, because the residue is buried, it is conceivable that the deficiency is caused by improper folding of the SH2 domain.

## Conclusions

The present structure provides the first view of the Jak2 FERM-SH2 module and an important point of comparison for the Tyk2 FERM-SH2, the only other Jak family member for which a structure of this region is available. The overall fold and interdomain interactions are closely preserved, and we expect that they are common to Jak1 and Jak3 as well. Prior structure-function studies have been limited by a lack of structural information for this region of Jak family members. The Jak2 structure presented here, as well as the Tyk2 structure in complex with an IFNAR1 fragment, will facilitate more focused dissection of the role of the FERM-SH2 in receptor recognition. Clearly, additional structures of these and other Jak family members in complex with their cognate receptors will be required to understand how specificity is maintained. Additionally, further structural and biochemical studies are required to understand if and how the FERM-SH2 module may interact with the pseudokinase and kinase domains to participate in regulation of kinase activity.

## Supporting Information

S1 Fig Oligomeric association of molecules in the Jak2 FERM-SH2 structure. (A) The crystallographic asymmetric unit of the Jak2 FERM-SH2 structure, with each of the eight Jak2 molecules colored differently. The “top” view is rotated 90° from the “side” view. (B) SEC/MALS analysis of Jak2 FERM-SH2. Molar mass (red trace) and differential refractive index for the Jak2 peak (blue trace) are plotted against retention time over a Superdex 200 10/300 GL column. The measured molecular weight was 61.5 kDa (+/- 8%), indicating monomeric FERM-SH2 (actual molecular weight 57.7 kDa).
(TIF)

S2 Fig Phase improvement by NCS averaging. (A) Electron density (yellow mesh) calculated following molecular replacement and rigid body refinement. (B) Electron density (purple mesh) calculated following density modification using 8-fold NCS averaging. The Cα trace of the molecular replacement search model is displayed as magenta lines. Figures were generated using Coot.
(TIF)

S3 Fig Difference distance matrix plot, Jak2 FERM SH2 vs. Tyk2 FERM-SH2. The difference distance matrix of Tyk2 is subtracted from that of Jak2, with the difference in angstroms between equivalent Cα positions plotted according to the indicated color scale. Difference distance matrix analysis and plot performed using DDMP (P.J. Fleming).
(TIF)
