# Supplementary figures and images for: Crystal Structure of the FERM-SH2 Module of Human Jak2
Source: PLoS One. 2016 May 26;11(5):e0156218. doi: 10.1371/journal.pone.0156218 (PMC4881981; doi:10.1371/journal.pone.0156218)

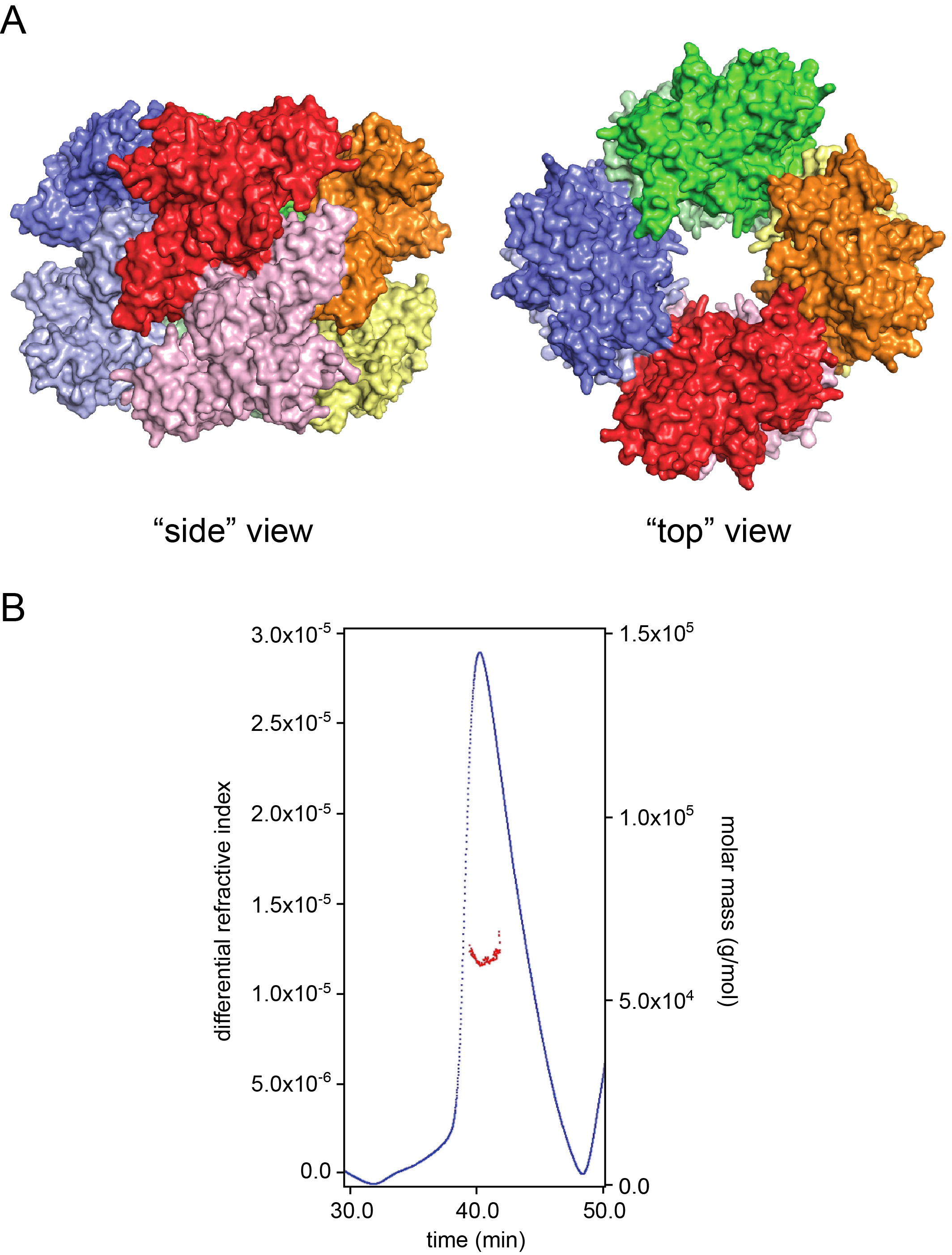

Supplement: S1 Fig — (A) The crystallographic asymmetric unit of the Jak2 FERM-SH2 structure, with each of the eight Jak2 molecules colored differently. The “top” view is rotated 90° from the “side” view. (B) SEC/MALS analysis of Jak2 FERM-SH2. Molar mass (red trace) and differential refractive index for the Jak2 peak (blue trace) are plotted against retention time over a Superdex 200 10/300 GL column. The measured molecular weight was 61.5 kDa (+/- 8%), indicating monomeric FERM-SH2 (actual molecular weight 57.7 kDa). (TIF) [file pone.0156218.s001.tif]

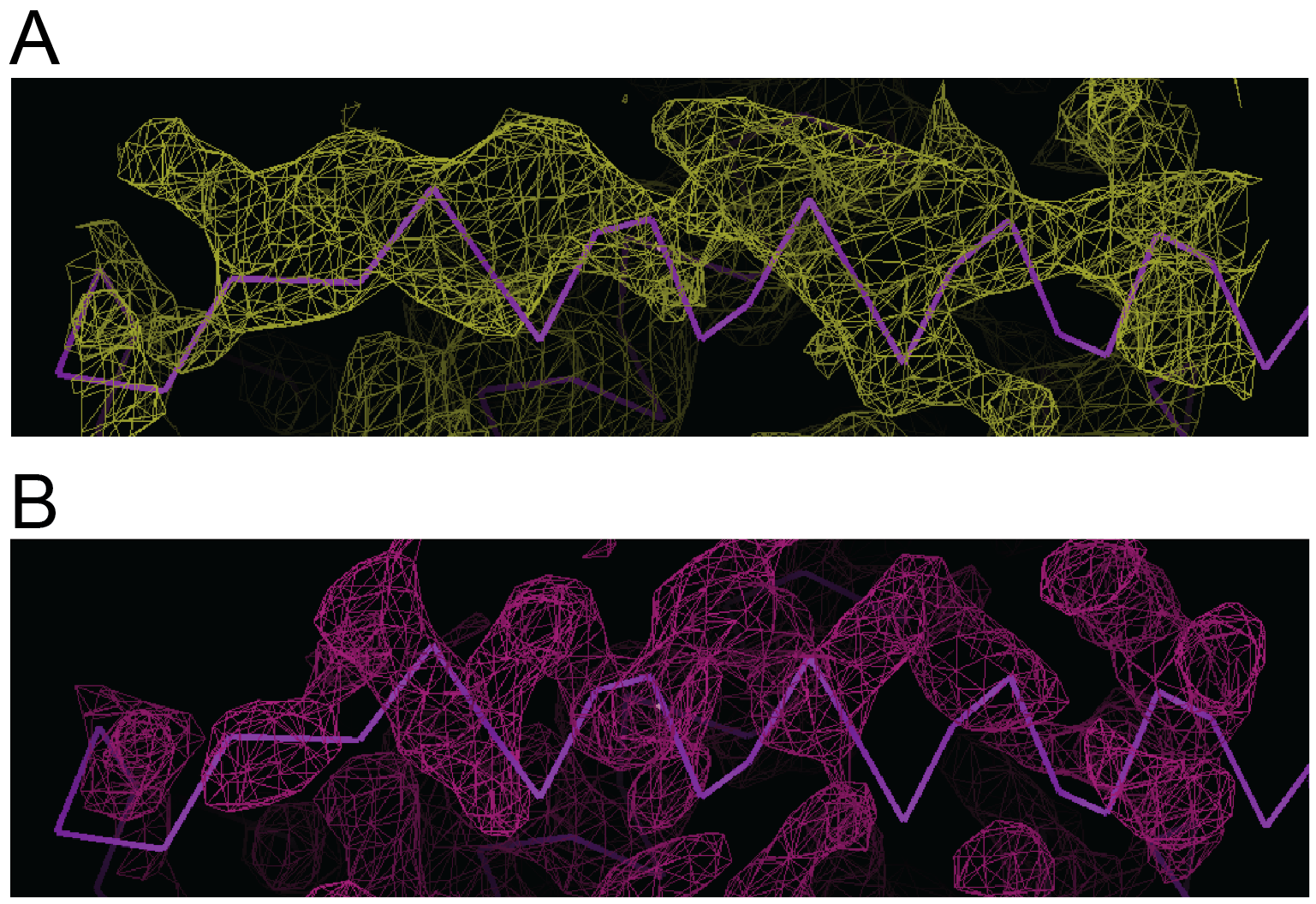

Supplement: S2 Fig — (A) Electron density (yellow mesh) calculated following molecular replacement and rigid body refinement. (B) Electron density (purple mesh) calculated following density modification using 8-fold NCS averaging. The Cα trace of the molecular replacement search model is displayed as magenta lines. Figures were generated using Coot. (TIF) [file pone.0156218.s002.tif]

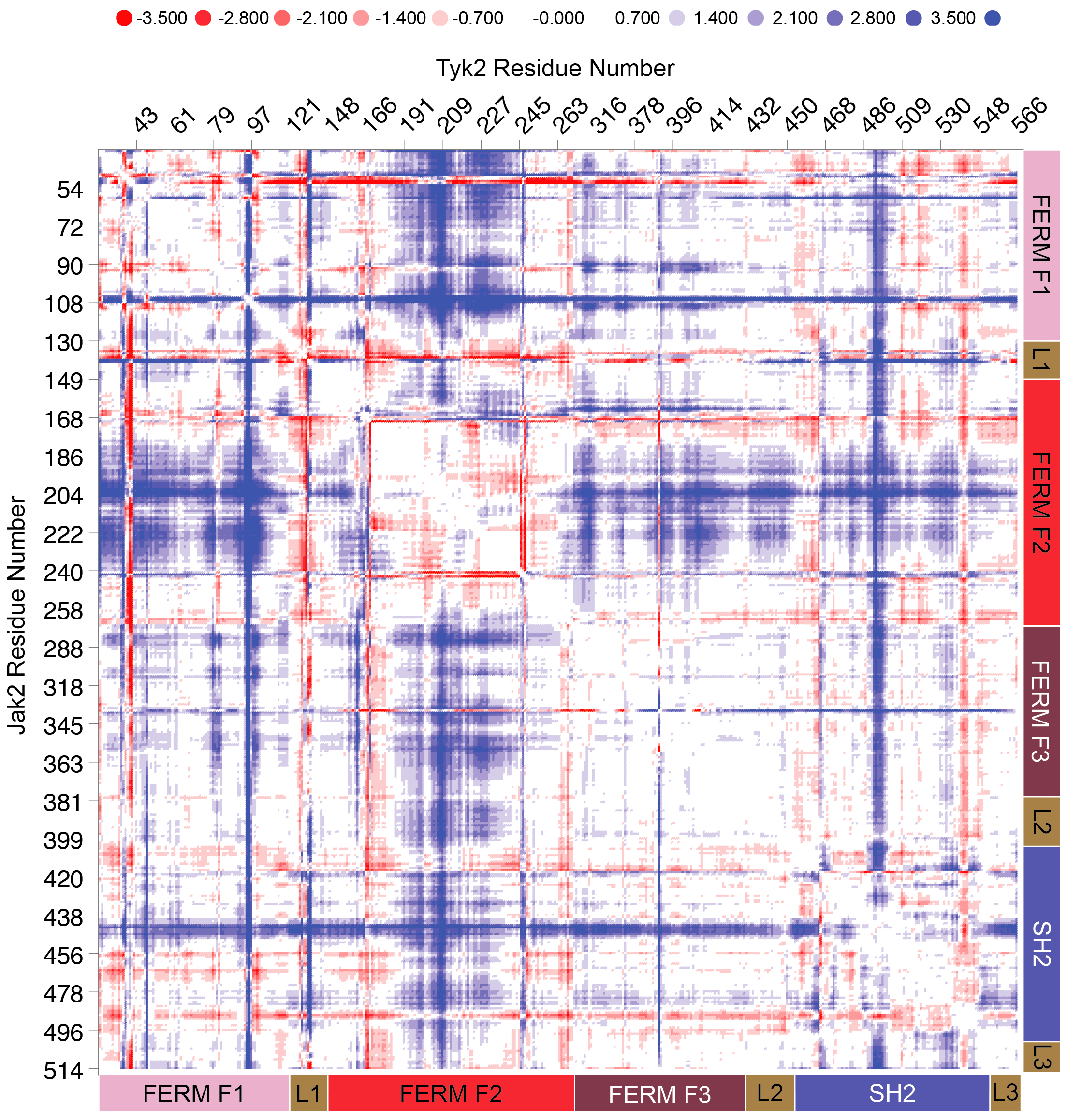

Supplement: S3 Fig — The difference distance matrix of Tyk2 is subtracted from that of Jak2, with the difference in angstroms between equivalent Cα positions plotted according to the indicated color scale. Difference distance matrix analysis and plot performed using DDMP (P.J. Fleming). (TIF) [file pone.0156218.s003.tif]
